# Self-harm and suicidality experiences of autistic and non-autistic adults in midlife and old age

**DOI:** 10.1186/s13229-025-00693-x

**Published:** 2025-11-25

**Authors:** Sophie Roper, Rebecca Charlton, Francesca Happé, Gavin R. Stewart

**Affiliations:** 1 Social, Genetic and Developmental Psychiatry Centre, Institute of Psychiatry, Psychology & Neuroscience, King’s College London, London, SE5 8AF UK; 2 Department of Psychology, Goldsmiths University of London, London, SW14 6NW UK

**Keywords:** Autism, Self-harm, Suicidality, Midlife, Old age

## Abstract

**Background:**

Suicide has been reported as a leading cause of premature death in autistic populations. Additionally, risk of suicidality is often found to increase with age in the general population. Despite this, suicidality has seldom been explored in autistic populations in midlife and old age. This study investigates the self-reported prevalence of self-harm and suicidality in autistic people in midlife and old age compared to an age- and gender-ratio comparable non-autistic group.

**Methods:**

In total, 388 participants (autistic *n* = 222, 44% men) aged 40–93 years (mean = 60.9 years) from the AgeWellAutism study completed questionnaires related to experiences of suicidal ideation, self-harming thoughts, deliberate self-harm, and suicidal self-harm. Group, gender and age differences were examined in chi-square and linear regression analyses.

**Results:**

The autistic group reported significantly higher rates of suicidal ideation, self-harming thoughts, deliberate self-harm, and suicidal self-harm than the non-autistic comparison group. When considering gender differences in the autistic group (but not the non-autistic group due to limited sample size), autistic women reported significantly higher rates of suicidal ideation and suicidal self-harm compared to autistic men; no other gender differences were found. When considering age differences, autistic people in old age were more likely to have had thoughts of self-harm, to have deliberately self-harmed, and to have experienced suicidal self-harm than autistic people in midlife.

**Limitations:**

The AgeWellAutism study is a cross-sectional convenience sample that relies on self-report. Survivor bias may also influence findings, as the study design would exclude those who have died by suicide, potentially leading to an underestimation of suicidality.

**Conclusions:**

Autistic adults may be particularly susceptible to experiences of self-harm and suicidality in midlife and old age, particularly autistic women. Additionally, autistic people in old age were also more likely to experience suicidality (including recent experiences) than autistic people in midlife. These findings highlight the urgent need for targeted suicide prevention strategies and mental health interventions for autistic adults in midlife and old age, particularly autistic women and older people.

**Supplementary Information:**

The online version contains supplementary material available at 10.1186/s13229-025-00693-x.

## Introduction

Suicidality – an umbrella term for suicidal ideation, suicide plans, self-harming with the intention of suicide, and death by suicide – is a global public health concern, with suicide accounting for 11.4 deaths per 100,000 people in the UK [1]. While women are more often found to engage in self-harming behaviours (whether with suicidal or non-suicidal intent), death by suicide occurs more commonly among men than women at a ratio of 3.5 to 1, with men often using more lethal methods of suicide [2]. While death by suicide has been recorded at all ages, high incidence rates of suicide in the UK are often found in midlife (age 40–64 years), which then peak in old age (64 years and older) [1]. Many factors have been attributed to this spike in later life, such as age-related change to health and cognition, and social factors [3]. Despite this peak in incidence of suicidality in midlife and old age, few studies have explored whether populations known to be at high risk of suicide, for example autistic people, have increased rates of suicidality in this later age-range.

Autism is a lifelong neurodevelopmental condition affecting approximately 1% of the global population [4]. Characterised by differences in social communication and rigid-repetitive behaviours and interests, autism plays a significant role in shaping individuals’ experiences throughout their lives [5, 6]. Autistic adults often experience poorer physical and mental health, lower education rates, lower employment rates, higher rates of traumatic events, more sleep disturbances, and lower overall quality of life [7–15]. These negative outcomes may be relevant to the high rates of suicidality compared to non-autistic people [16]. Multiple studies, including those using extensive population registers (from countries such as Australia, Korea, France, Sweden, Taiwan and the UK) and genetically informed designs (e.g., UKBioBank), have consistently shown that autistic people are at a heightened risk for suicidal ideation [16–18], non-suicidal self-harm [18–21], suicidal self-harm [18, 22–24], and death by suicide [23, 25–27]. Estimates pooled from 36 primary studies involving over 46,000 autistic people (age means = 10 to 42 years) suggest that approximately 34% of autistic people have experienced suicidal ideation, 22% have considered plans for suicide, and 24% have harmed themselves with the intention of suicide [16]. Suicide has also been found to be a leading cause of premature death in autistic people (ranked third in a Swedish population-based study, accounting for 12% of deaths in a sample of autistic decedents [25], with autistic people being up to nine times more likely to die by suicide compared to the general population [28]. Additionally, unlike in the general population, death by suicide is found to be higher in autistic women than autistic men, highlighting an important consideration for how suicidality is identified and risk assessed autistic populations [27].

When considering suicidality in autistic populations in midlife and old age, information is sparse. Despite population estimates suggesting that there are upwards of 250,000 autistic people in the UK over the age of 50 [29], research on middle-aged and older autistic adults accounts for only 0.4% of indexed autism research since 1980 [30, 31]. To the authors’ knowledge, only one study to date has specifically explored suicidality in midlife and old age in relation to autism. Stewart et al. (2023) [32] utilising a dimensional trait-based approach to account for high rates of underdiagnosis in midlife and older populations [29], showed that adults with high autistic traits (*n* = 276, aged 50–81, 31% men) self-reported significantly higher rates of suicidal ideation (72.7% vs. 29.3%), any deliberate self-harm (19.9% vs. 4.7%) and suicidal self-harm (12.7% vs. 2.5%) when compared to age and gender-matched low autistic trait individuals (*n* = 10,495); these higher rates represented a five-to-six-fold increase in the likelihood of suicidality for the high autistic trait participants. Importantly, this pattern remained when accounting for symptoms of depression.

Despite growing research on autism and suicidality, there remains a significant gap in our understanding of how these issues manifest and change throughout the lifespan, especially in mid-to-late adulthood. The current study seeks to replicate the findings of Stewart et al.’s (2023) [32] study. Stewart and colleagues examined a general population sample of middle-aged and older people endorsing high vs. low autistic traits. Our study will examine a similarly aged sample of adults who have a diagnosis of autism or who self-identify as autistic vs. a group of non-autistic adults. This replication will utilise the same methodological approach, employing the same self-report measure of self-harm and suicidality, and the same analytic approach. Furthermore, our study also seeks to extend Stewart et al.’s study by examining age-related differences in rates of self-harm and suicidality. In our study, we hypothesise that a similar pattern of results will be found in our middle-aged and older adult sample who have an autism diagnosis/self-identify as autistic. Namely, (1) autistic adults will self-report higher rates of self-harm and suicidality (i.e., suicidal ideation, self-harming thoughts, any deliberate self-harm, methods of self-harm, and suicidal self-harm) than age-and-gender comparable matched non-autistic adults. Gender differences will be found, with (2) autistic women reporting higher rates of self-harm and suicidality than autistic men. And finally, age differences will also be found, with (3) autistic adults aged 65+ (i.e., older adults) reporting more experiences of suicidal self-harm than autistic adults aged 40–64 years (i.e., middle-aged adults).

## Method

### Study design

This study uses cross-sectional data from the first wave of the ‘AgeWellAutism’ study, conducted in Spring 2019. The AgeWellAutism study is an online survey investigating ageing on the autism spectrum (outlined in Stewart et al., (2024) [33]. In brief, the content of the AgeWellAutism study was steered by 12 middle-aged and older autistic adults prior to launch, with recruitment being conducted through social media platforms (i.e., Twitter, Reddit, Facebook), participant databases managed by the author and Autistica’s Research Network, and adverts in community centres and older adult residential communities. An incentive of entry into a raffle for a £20 Amazon UK gift voucher was offered. Inclusion criteria were being 40 years of age or above, having access to an internet-enable device, and being able to read and type in English. The study had no specific exclusion criteria. Full ethical approval was granted by the PNM Research Ethics Subcommittee at King’s College London (HR-18/19–10941).

### Participants

In total, 502 completed surveys were recorded, with 70 responses being removed due to suspected spam (i.e., very short completion times and/or irregular answers to open-text boxes). For the current study, a further 40 responses were excluded as these individuals opted to skip the self-harm and suicidality questions. An additional four non-binary/trans/agender participants were also omitted due to the small sample size. This resulted in a final sample of 388 participants aged 40–93 years. Participants who disclosed that they either had an autism diagnosis (*n* = 211) or self-identified as autistic (*n* = 11) formed an autistic group (*n* = 222); both subgroups had comparable autistic trait scores (*t*(1,223) = 1.63, *p* = .104). The autistic group were asked when they received their autism diagnosis/ began to identify as autistic; responses ranged from as recently as the year of survey completion to 43 years ago (mean years since diagnosis = 9.4 years), with nine (4.1%) diagnosed in childhood (i.e., prior to 18 years of age). The remaining participants formed the non-autistic group (*n* = 166). The autistic and non-autistic groups were comparable on age (autistic mean age = 60.9 years; non-autistic mean age = 60.6 years) and gender ratio (autistic group men = 44.1%; non-autistic group men = 50.6%). For the age group analyses, 131 autistic people were in the midlife group (59%, ages 40–64) and 88 were in the old age group (41%, aged 65+). Both groups were comparable in education level and in country of residence, with around 98% living in the UK. However, the autistic sample self-reported lower employment rates and a higher likelihood of living with non-marital family members. No data on socio-economic status and race/ethnicity was collected. See Table 1 for demographic characteristics of the autistic and non-autistic groups.

**Table 1:** Demographic characteristics of autistic and non-autistic groups

	Autistic (*n* = 222)	Non-Autistic (*n* = 166)	Group Difference	Effect Size
**Age (years)**				
M (SD)	60.89 (12.84)	60.55 (13.58)	*t(386)* = 0.247 *p =* .805^n/s^	*d* = 0.03 [-0.23, 0.18]
[95% CI]	[59.18, 62.59]	[58.47, 62.64]		
Range	40–91	40–93		
Midlife: Old Age (n)	131: 88 (41%:59%)	-		
**Gender**				
N men: women	98: 124	84: 82	χ^2^ = 1.59, *p* = .207^n/s^	*v* = 0.06
%	44.1%: 55.9%	50.6%: 49.4%		
**Living Situation**				
Spouse or partner	85 (38.3%)	79 (47.6%)	χ^2^ = 3.37, *p =* .066^n/s^	*v* = 0.09
Children	62 (27.9%)	39 (23.5%)	χ^2^ = 0.97, *p* = .325^n/s^	*v* = 0.05
Sibling	24 (10.8%)	0 –	χ^2^ = 19.13, *p* < .001***	*v* = 0.22
Parent	19 (8.6%)	7 (4.2%)	χ^2^ = 2.86, *p* = .09^n/s^	*v* = 0.09
Other family member	7 (3.2%)	0 –	χ^2^ = 5.33, *p* = .021*	*v* = 0.12
Roommate/friend	20 (9.0%)	12 (7.2%)	χ^2^ = 0.40, *p =* .528^n/s^	*v* = 0.03
Supported Housing	38 (17.1%)	20 (12.0%)	χ^2^ = 1.92, *p* = .166^n/s^	*v* = 0.07
Alone/Independently	41 (18.5%)	57 (34.3%)	χ^2^ = 12.67, *p <* .001***	*v* = 0.18
**Education Level**				
No formal qualifications	23 (10.4%)^ǂ^	4 (2.4%)^ǂ^	χ^2^ = 16.97 *p* = .002**	*v* = 0.21
School to 16	50 (22.5%)	27 (16.3%)		
School to 18	61 (27.5%)^ǂ^	65 (39.2%)^ǂ^		
Undergraduate	54 (24.3%)	51 (30.7%)		
Postgraduate	34 (15.3%)	19 (11.4%)		
**Employment Status**				
Employed	69 (31.1%)^ǂ^	87 (52.4%)^ǂ^	χ^2^ = 35.04 *p* < .001***	*v* = 0.30
Unemployed	24 (10.8%)^ǂ^	3 (1.8%)^ǂ^		
Unable to work due to health	24 (10.8%)^ǂ^	2 (1.2%)^ǂ^		
Retired	105 (47.5%)	74 (44.6%)		
**Autism Diagnosis/Identification**				
Diagnosed	211 (95.1%)	-	-	
Self-Identified	11 (4.9%)	-	-	
**Years since Autism Diagnosis/Identity**				
M (SD)	9.42 (7.66)	-	-	-
Range (years)	0–43	-	-	-
**Autism Traits** (max score = 42) M (SD)	32.74 (7.53)	2.95 (4.07)	*t*(386) = 49.96, *p* < .001***	*d* = 4.74 [4.07, 7.53]

Note. *N* = 388. Midlife = 40–64 years, Old Age = 65+. *d* = Cohen’s *d. v* = Cramer’s *v.* *p < .05, **p < .01. ***p < .001

### Materials

*Demographic Characteristics –* Participants provided detailed self-reported demographic information, including age, gender, highest educational attainment, employment status, who they live with, and country of residence.

Autism Diagnoses/Self-identification and Autistic Traits - Participants were asked whether they had an autism diagnosis or if they self-identified as autistic. If yes, they were then asked how many years had passed since their diagnosis or they began to self-identify.

Additionally, the Ritvo Autism and Asperger Diagnostic Scale (RAADS-14) [34] was used to descriptively explore autistic trait endorsement in each group, to ensure the diagnosed and self-identified autistic participants could be combined into one group. The RAADS-14 is a 14-item questionnaire that explores autistic traits related to mentalising difficulties, sensory reactivity, and social anxiety. Scores range from 0 to 42, with a score ≥ 14 having a sensitivity of 97% and specificity of 46%-64% for identifying autism. The RAADS-14 has demonstrated satisfactory psychometric properties [35]. In the current study, the RAADS-14 showed very good internal consistency in the autistic group (Cronbach’s *a =* 0.887), and good consistency in the non-autistic group (Cronbach’s *a =* 0.744).

*Self-Harm and Suicidality –* Experiences of suicidality were measured using the 8-item self-report UK BioBank Self-Harm and Suicidality Questionnaire [32]. These questions were presented on an opt-in basis, with the option to skip questions at any point during the block. Suicidal ideation was assessed by asking participants if they: (i) had felt that life was not worth living. For thoughts of harm, participants were asked whether they: (ii) had contemplated harming themselves, and whether they: (iii) had contemplated harming themselves in the last 12 months. For deliberate harm, frequency of harm, and types of harm, participants were asked: (iv) if they had ever deliberately harmed themselves, whether they meant to end their life or not; (v) how many times they had deliberately harmed themselves; (vi) if they had deliberately harmed themselves within the last 12 months; and (vii) the types of harming behaviours used. For suicidal harm, participants were asked if they: (viii) had deliberately taken an overdose or harmed themselves with the intention to end their life. For the complete list of questions and scoring matrix see supplementary materials.

The UK BioBank Self-Harm and Suicidality questionnaire has been used in samples previously, including in older adults with high autistic traits [32]. In the current study, the scale has very good internal consistency in the autistic (Cronbach’s *a* = 0.807) and non-autistic (Cronbach’s *a* = 0.849) groups.

*Depression* – Symptoms of depression were measured using the Patient Health Questionnaire (PHQ-8) [36]. The PHQ-8 is an eight-item questionnaire with a 4-point scale which asks the participant to report whether they have been bothered by a range of problems over the past two weeks such as anhedonia, low mood, sleep problems, fatigue, poor appetite or weight change, concentration difficulty, and psychomotor disturbance. The PHQ-8 omits the suicidal ideation question included in the nine-item version of the measure (PHQ-9). Using the conventional cut-off score of ≥ 10, the PHQ-9 has a sensitivity of 88% and a specificity of 88% for major depressive disorder. The PHQ-9 has been validated for use in autistic adults [37].

### Data analysis

The current study using the AgeWellAutism dataset (specifically hypotheses 1 and 2) was pre-registered prior to analyses being conducted (https://osf.io/qv23s). *Hypothesis 3 (i.e.*,* age-differences) was added as a post-hoc analysis and not included in the original pre-registration.* All statistical analyses were performed using SPSS (v29.0.2.0; IBM Corp., 2023).

Demographic variables and autism-related information was analysed with t-tests (continuous variables) and chi-squared tests (χ²; categorical variables). To address hypothesis 1, χ² analyses were conducted to investigate group differences (autistic vs. non-autistic) in rates of self-harm and suicidality experiences. A series of linear regressions (with suicidality items as outcomes, and autism group and depression scores as predictors) were conducted to examine whether any observed group differences in suicidality were solely a function of worse depression symptomology. To address hypothesis 2, additional layered χ² analyses were performed to examine differences in self-harm and suicidality scores by gender (men vs. women) and group (autistic vs. non-autistic). To address hypothesis 3, χ² analyses were conducted to investigate age group differences (midlife (40–64 years) vs. old age (65 + years)) in rates of self-harm and suicidality experiences in the autistic group; these analyses were not conducted in the non-autistic group due to small group sizes. Exploration of these age group differences was added due to the lack of research that specifically focuses on autistic people in old age; as health services often have specialist services for people aged over 65, we opted to stratify our sample into midlife (ages 40–64) and old age (aged 65+) subgroups. Across these hypotheses, adjusted residual values were examined to identify significant differences in ratings or proportions, and odds ratios (OR) were calculated with ‘yes’ options collapsed.

Multiple comparisons were accounted for by using the False Discovery Rate method (FDR) [38], using an initial alpha level of 0.050. The FDR procedure was applied to all p-values, and adjusted alpha values were assigned based on the rank of the *p*-values; final FDR adjusted critical value being 0.044.

Our post-FDR power calculation indicated a total sample size of 212 participants (i.e., at least 54 per subgroup) was required to detect medium effect sizes with the FDR-adjusted alpha level of 0.044 while maintaining 95% statistical power. Our original a priori calculation (with an alpha level of 0.050, prior to FDR adjustment) required at least 52 per subgroup while maintaining 95% statistical power.

## Results

### Group differences in self-harm and suicidality

#### Suicidal ideation

The autistic group self-reported significantly higher rates and higher frequencies of suicidal ideation compared to the non-autistic group (χ2 = 129.84, *p* < .001). In total, 73.0% of the autistic group had experienced suicidal ideation (13.2% once, 59.8% more than once) in comparison to 25.9% of the non-autistic group (21.7% once, 4.2% more than once). When collapsing ‘yes’ responses, the autistic group were almost eight times more likely than the non-autistic group to have experienced suicidal ideation at least once (OR = 7.85). The pattern of results remained when accounting for the influence of current depression symptoms (R^2^ = .346, F = 105.54, *p* < .001; depression *b* = .25, autism group *b* = .41). See Table 2.

**Table 2:** Self-reported prevalence rates of self-harm and suicidality in the autistic and Non-Autistic groups

	Autistic (*n* = 222)	Non-Autistic (*n* = 166)	Group difference	Effect size	Odds ratio
**Suicidal Ideation - Many people have felt that life is not worth living. Have you felt that way?**					
No	59 (26.9%)^ǂ^	123 (74.1%)^ǂ^	χ^2^ = 129.84, *p* < .001***	*v* = 0.58	7.85 [4.97, 12.41]
Yes, once	29 (13.2%)^ǂ^	36 (21.7%)^ǂ^			
Yes, more than once	131 (59.8%)^ǂ^	7 (4.2%)^ǂ^			
**Thoughts of Harm - Have you contemplated harming yourself (for example by cutting**,** biting**,** hitting yourself or taking an overdose)?**					
No	73 (33.5%)^ǂ^	124 (74.7%)^ǂ^	χ^2^ = 85.67, *p* < .001***	*v* = 0.47	5.90 [3.77, 9.25]
Yes, once	29 (13.3%)	27 (16.3%)			
Yes, more than once	116 (53.2%)^ǂ^	15 (9.0%)^ǂ^			
**Thoughts of Harm (Recent) - Have you contemplated harming yourself in the last 12 months?**					
No	123 (56.4%)	157 (94.6%)	χ^2^ = 69.48, *p* < .001***	*v* = 0.43	13.47 [6.54, 27.77]
Yes	95 (43.6%)	9 (5.4%)			
**Any Deliberate Harm - Have you deliberately harmed yourself**,** whether or not you meant to end your life?**					
No	99 (45.0%)	144 (86.7%)	χ^2^ = 69.29, *p* < .001***	*v* = 0.43	7.86 [4.67, 13.26]
Yes	121 (55.0%)	22 (13.3%)			
**Frequency of Harm – If yes**,** how many times have you harmed yourself?**					
Once	18 (14.9%)^ǂ^	13 (54.2%)^ǂ^	χ^2^ = 22.62, *p* < .001***	*v* = 0.40	-
Twice	13 (10.7%)^ǂ^	6 (25.0%)^ǂ^			
Three or more times	90 (74.4%)^ǂ^	5 (20.8%)^ǂ^			
**Any Deliberate Harm (Recent) - Have you harmed yourself in the last 12 months**,** whether or not you meant to end your life?**					
No	181 (83.0%)	160 (96.4%)	χ^2^ = 16.91, *p* < .001***	*v* = 0.21	5.41 [2.24, 13.25]
Yes	37 (17.0%)	6 (3.6%)			
**Suicidal Harm - Have you deliberately taken an overdose or harmed yourself with the intention to end your life?**					
No	174 (79.8%)	160 (96.4%)	χ^2^ = 22.84, *p* < .001***	*v* = 0.24	6.74 [2.80, 16.25]
Yes	44 (20.2%)	6 (3.6%)			

Note. ^ǂ^ Adjusted residual indicates proportional difference. Odds ratios calculated with yes/frequency options grouped. *p < .05, **p < .01. ***p < .001

#### Self-harming thoughts

The autistic group self-reported significantly higher rates and higher frequencies of self-harming thoughts compared to the non-autistic group (χ^2^ = 85.67, *p* < .001). In total, 66.5% of the autistic group (13.3% once, 53.2% more than once) had experienced these thoughts, in comparison to 25.3% of the non-autistic group (16.3% once, 9.0% more than once). When collapsing ‘yes’ responses, the autistic group were nearly six times more likely than the non-autistic group to have experienced self-harming thoughts at least once (OR = 5.90). The pattern of results remained when accounting for the influence of current depression symptoms (R^2^ = .277, F = 75.14, *p* < .001; depression *b* = .31, autism group *b* = .28). See Table 2.

Additionally, the autistic group self-reported significantly higher rates of self-harming thoughts within the last 12 months compared to the non-autistic group (χ^2^ = 69.48, *p* < .001). In total, 43.6% of the autistic group had contemplated self-harm recently, compared to 5.4% of the non-autistic group. The autistic group were thirteen times more likely than the non-autistic group to have experienced self-harming thoughts in the last year (OR = 13.47). The pattern of results remained when accounting for the influence of current depression symptoms (R^2^ = 0.265, F = 70.68, *p* < .001; depression *b* = 0.37, autism group *b* = 0.20).

#### Any deliberate self-harm

The autistic group self-reported significantly higher rates of any deliberate self-harm compared to the non-autistic group (χ^2^ = 69.29, *p* < .001). In total, 55.0% of the autistic group had deliberately self-harmed (whether suicidal or non-suicidal), compared to 13.3% of the non-autistic group. The autistic group were nearly eight times more likely than the non-autistic comparison group to have deliberately self-harmed (whether suicidal or non-suicidal) at least once (OR = 7.86). Among those who had engaged in any deliberate self-harm, the autistic group reported significantly higher frequencies of self-harm incidents than the non-autistic group (χ^2^ = 22.62, *p* < .001). Of those who had engaged in any deliberate self-harm, 85.1% of the autistic group had two or more incidents of self-harm compared to 45.8% of the non-autistic group. The patterns of results remained when accounting for the influence of current depression symptoms (R^2^ = 0.279, F = 76.09, *p* < .001; depression *b* = 0.37, autism group *b* = 0.21). See Table 2.

Additionally, the autistic group self-reported significantly higher rates of deliberate self-harm within the last 12 months compared to the non-autistic group (χ^2^ = 16.91, *p* < .001). In total, 17.0% of the autistic group had deliberately self-harmed recently, compared to 3.6% of the non-autistic group. The autistic group were five times more likely than the non-autistic comparison group to have deliberately self-harmed in the last year (OR = 5.41). However, when accounting for the influence of current symptoms of depression, autism group was no longer a significant predictor of recent self-harm (R^2^ = 0.112, F = 24.88, *p* < .001; depression *b* = 0.32, autism group *b* = 0.03). See Table 2.

#### Methods of deliberate self-harm

Among those who had engaged in any deliberate self-harm, no statistically significant differences were found, with comparable rates of self-harming methods being reported by the autistic and non-autistic groups. Additionally, when considering the number of methods of deliberate self-harm that participants used, no statistical differences between the autistic and non-autistic groups were found. See Table 3 for frequencies of self-harming behaviours between groups.

**Table 3:** Self-reported types of harm used by those who have self-harmed

	Autistic (*n* = 116)	Non-autistic (*n* = 22)	Group difference	Effect size
**Types of Harm – Have you done any of the following to harm or endanger yourself?**				
Stopping prescribed medication	21 (18.1%)	3 (13.6%)	χ^2^ = 0.28, *p* = .612^n/s^	*v* = 0.04
Swallowing dangerous objects or products	27 (22.7%)	1 (4.5%)	χ^2^ = 3.84 *p* = .050^n/s^	*v* = 0.17
Ingesting alcohol or recreational/illicit drug	28 (23.7%)	3 (13.6%)	χ^2^ = 1.10, *p* = .295^n/s^	*v* = 0.09
Ingesting a medication more than the normal dosage	59 (49.6%)	11 (50.0%)	χ^2^ = 0.01, *p* = .971^n/s^	*v* < 0.01
Self-injury (e.g., cutting, scratching, hitting)	74 (61.7%)	10 (45.5%)	χ^2^ = 2.02, *p* = .155^n/s^	*v* = 0.12
**Number of Self-harming methods used**				
None of the above	15 (12.9%)	2 (1.7%)	χ^2^ = 5.13, *p* = .163^n/s^	*v* = 0.19
One	43 (37.1%)	11 (9.5%)		
Two	30 (25.9%)	8 (6.9%)		
Three or more	28 (24.1%)	1 (0.9%)		

Note. Participants could select multiple options, thus total does not equal 100%

#### Suicidal self-harm

The autistic group self-reported significantly higher rates of suicidal self-harm in comparison to the non-autistic group (χ^2^ = 22.84, *p* < .001). In total, 20.2% of the autistic group had engaged in suicidal self-harm, in comparison to 3.6% of the non-autistic group. The autistic group were nearly seven times more likely to engage in suicidal self-harm than the non-autistic group (OR = 6.74). The pattern of results remained when accounting for the influence of current depression symptoms (R^2^ = 0.101, F = 21.99, *p* < .001; depression *b* = 0.23, autism group *b* = 0.12). See Table 2.

When examining the overlap between suicidal ideation and suicidal self-harm, 26% of autistic individuals who had experienced suicidal ideation also engaged in suicidal self-harm, in comparison to 14% of non-autistic individuals. This difference was significant (χ^2^ = 43.42, *p* < .001) with a medium effect size (Cramer’s *v =* 0.34).

### Gender differences in self-harm and suicidality

When comparing the experience of autistic women to autistic men, our analyses indicated that autistic women self-reported significantly higher rates of suicidal ideation (women: 77.4% vs. men: 67.0%, respectively; χ^2^ = 9.78, *p* = .008), thoughts of self-harm (74.2% vs. 56.4%; χ^2^ = 9.64, *p* = .008), any deliberate self-harm (62.1% vs. 44.7%; χ^2^ = 6.54, *p* = .011) and suicidal self-harm (26.6% vs. 11.7%; χ^2^ = 7.38, *p* = .007) when compared to autistic men; however, no statistical differences were found between autistic women and autistic men in recent thoughts of self-harm or recent deliberate self-harm (whether suicidal or non-suicidal). When considering the frequency of these experiences, autistic women were found to have more frequent thoughts of suicidal ideation (8.9% once, 68.5% more than once) when compared to autistic men (18.6% once, 48.4% more than once), as well as more frequent of thoughts of harm (autistic women: 12.1% once, 61.1% more than once; vs. autistic men:14.9% once, 41.5% more than once), but not in the frequency of deliberate harm. Few gender differences were found in the non-autistic group, with only rates of suicidal ideation reaching statistical significance (non-autistic women: 32.9% vs. non-autistic men: 19.0%; χ^2^ = 8.80, *p =* .012). The patterns of results remained when accounting for the influence of current depression symptoms. See Supplementary Table 1.

For methods of self-harming behaviours, no statistical differences were found between autistic women and autistic men, with similar rates of each method of harm being used. This sub-group analysis was not conducted in the non-autistic group due to low endorsement of suicide experiences resulting in low statistical power. See Supplementary Table 2.

### Age differences in self-harm and suicidality in the autistic group

When comparing the experience of autistic people in midlife (ML; i.e., aged 40–64 years) and old age (OA; i.e., aged 65 + years), our analyses indicated that autistic people in old age self-reported significantly higher rates of self-harming thoughts (OA: 76.1% vs. ML: 60.0%; χ^2^ = 6.20, *p* = .043), any deliberate self-harm (OA: 70.5% vs. ML: 43.8%; χ^2^ = 14.98, *p* < .001), recent deliberate self-harm (whether suicidal or non-suicidal) (OA: 23.9% vs. ML: 12.3%), and suicidal self-harm (OA: 28.4% vs. ML: 14.6%; χ^2^ = 4.97, *p* = .026) when compared to autistic people in midlife; however, no statistical differences were found between autistic people in midlife and old age in suicidal ideation (ML: 68.7% vs. OA: 79.5%). %). When considering the frequency of these experiences, autistic people in old age were found to have more frequent thoughts of thoughts of harm (OA: 60.2% more than once, vs. ML: 48.5% more than once), but not in the frequency of suicidal ideation or deliberate harm. The patterns of results in thoughts of harm, any deliberate self-harm and suicidal self-harm remained when accounting for the influence of current depression symptoms, however, age group as a predictor of recent thoughts of harm and recent deliberate self-harm (whether suicidal or non-suicidal) were no longer statistically significant after controlling for depression. See Supplementary Table 3.

For methods of self-harming behaviours, autistic people in midlife were statistically more likely to self-injure through cutting, scratching or hitting compared to the autistic people in midlife (ML: 75.4% vs. OA 49.2%; χ^2^ = 8.71, *p* = .003), while the autistic people in old were more likely to self-injure by ingesting medications more than the norma dosage (OA: 59.7% vs. ML: 38.6%; χ2 = 5.28, *p* = .0.22) or by swallowing dangerous objects or products (OA: 30.6% vs. ML: 14.0%; χ^2^ = 4.67, *p* = .031). No other statistical differences were found. See Supplementary Table 4.

## Discussion

Using data from a sample of people in midlife and old age, this study provides evidence that middle-aged and older autistic adults self-report higher rates and frequencies of self-harm and suicidality than age- and gender-comparable non-autistic adults, including when accounting for current symptoms of depression. Some gender differences were observed, with autistic women reporting higher rates and frequencies of suicidal ideation and higher rates of suicidal self-harm than autistic men. Additionally, autistic adults in old age had higher rates of any deliberate self-harm, including suicidal self-harm, than autistic adults in midlife. These findings – which broadly replicate and extend Stewart et al.’s (2023) [32] results from a high autistic trait (not diagnosed) sample – contributes valuable information about suicidality in autistic people in midlife and old age, a demographic that has historically been overlooked.

The first key finding in the current study was that the autistic participants in midlife and old age self-reported significantly higher rates of suicidal ideation and self-harming thoughts than non-autistic comparisons The autistic group were also significantly more likely to have a higher frequency of these problems, with 60% having experienced suicidal ideation more than once. Our findings align with previous research, such as a UK-based clinical cohort study by Cassidy et al., (2014) [17], which reported that 66% (*n* = 243) of autistic adults had contemplated suicide. Additionally, comparable rates were also found in Stewart et al., (2023) [32], which found that 73% of middle-aged and older adults with high autistic traits had experienced suicidal ideation. While suicidal ideation and thoughts of self-harm were not uncommon in the non-autistic group, the autistic group were six to eight times more likely to have had these experiences, highlighting that suicidality is a pressing health issue in autistic populations.

When considering why rates and frequencies of suicidal ideation may be so high in autistic populations, mental health challenges likely play a key role. Autistic individuals show higher prevalence rates for nearly all mental health conditions compared to non-autistic counterparts [8, 9, 13]. Mental health problems are also of concern in the current study, with the autistic group being found to report significantly higher depression symptom scores than the non-autistic group. There is a growing body of research as to why autistic people often have more mental health problems than the general population (e.g., social isolation, social stigma, victimisation and trauma, rigid cognitive style, and poorer normative outcomes) [28, 39], which in turn may exacerbate feelings of hopelessness, which is a core feature of suicidal ideation and may lead to periods of suicidal crisis [19, 22, 40]. This interaction between autism, biopsychosocial risk factors, and suicidal ideation suggests a complex interplay of factors. While our current study did account for depression in our analyses (with the pattern of results remaining unchanged) further investigation of the influence of depression and other risk factors is needed.

Other key findings of this study were the significantly higher rates of any deliberate self-harm reported by the autistic group, where over half of the autistic participants had engaged in self-harm (whether suicidal or non-suicidal). Of those who had harmed, approximately three-quarters of the autistic group had deliberately self-harmed three or more times. Additionally, the middle-aged and older autistic adults in this study also self-reported higher rates of suicidal self-harm than non-autistic adults. The rates of these deliberate and suicidal self-harm experiences in the current study are higher than those documented in previous research. For example, Hirvikoski et al. (2020) [23] and Stewart et al. (2023) [32] report that 8–13% of autistic or high autistic trait adults experience suicidal self-harm, compared to 17% of autistic participants in the present study. These differences could be attributed to a range of factors, including variations in study design (i.e., the current study uses convenience sampling vs. Stewart et al. uses a large-scale longitudinal study of healthy ageing vs. Hirvikoski et al. uses population registers), in how participants are identified in each study (e.g., the current study uses self-reported diagnosed/self-identified autistic people vs. Stewart et al. uses a high autistic traits approach vs. Hirvikoski et al. uses autism diagnostic codes in health records) and also how suicidality is reported (e.g., the current study and Stewart et al. use self-report vs. Hirvikoski et al. uses self-harm diagnostic codes in health records). Also, the conceptualisations of self-harming behaviours could play a role; some of these behaviours (e.g., hitting, scratching) may be stimming behaviours that autistic people engage in for therapeutic reasons [41]. Finally, there may be stigma and biases in how people self-report behaviours related to suicidality [42].

When considering the high rates of self-harm and suicidal behaviours reported in the current study and other studies involving autistic people with a diagnosis, several factors may play an important role in why autistic people engage in non-suicidal and suicidal self-harm [43, 44]. For some autistic individuals, particularly autistic women who are more likely to engage in self-harming behaviours, non-suicidal self-harm may serve as a coping mechanism to regulate sensory overload or express unmet emotional needs [45]. This may result in acquired capability of suicide (i.e., due to increased pain tolerance, reduced fear of death, and familiarity with the mental rehearsal of suicide [46]. This acquired capability may explain why autistic women are more likely to die by suicide than autistic men. Furthermore, late autism diagnoses may also contribute to higher rates of any deliberate self-harm, as individuals with a late diagnosis may experience greater social difficulties, mental health issues, and frustration due to the delayed recognition of their condition [19, 33, 47–53]. Autistic people may also be at higher probability of other risk factors for suicide, such as poor normative outcomes [19, 40, 54]. These issues fit with the ‘Interpersonal Theory of Suicide [55], which emphasises contributory factors of perceived burdensomeness, and thwarted belongingness, alongside capability for suicide. Given our study found that the relationship between being autistic and experiencing suicidality persisted even when accounting for symptoms of depression, it points towards a complex interplay of precipitating factors that culminate in suicidal thoughts and behaviours. While suicidal ideation does not always lead to suicidal behaviour, the significantly higher rates of co-occurring ideation and self-harm in autistic adults highlights the need for interventions that have been specifically designed to support autistic people holistically, for example tailored mental health support, ensuring autistic people have opportunities for supported employment, as well as social opportunities to develop relationships with others.

Our gender-based analyses also yielded important information when considering the experiences of suicidality in autistic populations. The autistic women in midlife and old age in our current study reported almost three times higher rates of self-harm and suicidality than the autistic men. This pattern of results mirrors that in the general population. When considering why autistic women may have such high rates of suicidality compared to autistic men, increased masking/camouflaging, later diagnosis, co-occurring mental health conditions, and societal pressures may disproportionately affect autistic women [56]. Additionally, acquired capability may play a role in autistic women engaging in suicidal self-harm [46]. These factors may contribute to the feelings of thwarted belongingness and perceived burdensomeness that are central to suicidality according to the interpersonal theory of suicide, which has been applied to the general and autistic populations [46, 55, 57, 58]. Previous research has consistently found similar gender disparities to our study, with autistic women at higher risk for suicidal thoughts and behaviours compared to autistic men [19, 27]. Taken together, these gender differences highlight the importance of adopting gender-sensitive approaches in both clinical practice and research.

Finally, our age-related analyses also provided important insight into potential vulnerabilities of autistic adults in old age (i.e., over 65 years old). Older autistic adults were found to report higher rates of self-harming thoughts, any deliberate self-harm, and suicidal self-harm when compared to autistic adults in midlife (i.e., 40–64 years old). While our questions often captured lifetime experiences (which likely increase with age), higher rates of recent self-harm were also found to be reported by the older autistic participants. These findings suggest that age-specific factors contribute to heightened self-harm risks in older populations, potentially due to increased social isolation [33, 48] and barriers to adequate healthcare support [59], warranting age-tailored interventions. Furthermore, the middle-aged autistic adults were more likely to self-injure through cutting, scratching, or hitting, while the older autistic adults were more likely to ingest medication overdoses or other dangerous objects/products indicating variations in self-harm methods across age groups. This highlights the importance of understanding age-related differences in clinical settings to better support autistic individuals throughout their lifespan.

The findings of the current study have significant clinical implications. While suicidality cannot be directly ‘treated’, interventions can address underlying risk factors. There is a clear need for tailored screening tools, gender-sensitive approaches, and age-appropriate interventions for autistic adults. Promoting social connection, mitigating perceived burdensomeness, and reducing stigma should be key components of suicide prevention strategies. Implementation of the 2009 Autism Act [60] and enhanced professional training for adult service providers remains crucial. Training is essential to equip providers to support the complex needs of the often-underserved older autistic population [61]. Future research priorities should include longitudinal studies to track changes in suicidality over time and development of comprehensive assessment tools [62]. Additionally, future studies should examine the experiences of autistic people from gender minorities (e.g., trans and non-binary people), a group also known to be vulnerable to suicidality [63, 64]. Finally, autistic people must be consulted when studying suicidality, to ensure the studies are conducted in a way sensitive to their experiences and to not cause unforeseen harm.

### Strengths and limitations

When contextualising the findings of the current study, several strengths and limitations are worth considering. For strengths, this study conducted extensive PPI interviews and project steering, which ensured topic relevance and accessibility. The breadth of survey topics likely reduced recruitment bias toward those with experiences of suicidality, increasing the generalisability of our findings. Additionally, diverse recruitment methods (e.g., online adverts, notices shared through large and well-established autism research networks, community centre notices) enhanced sample representativeness and includes older people who are potentially not actively using the internet and social media. Our sample was also comparable on key demographic factors, with almost half of the participants being men and the sample including those in later life (i.e., over the age of 80).

However, limitations must be acknowledged. The AgeWellAutism study was conducted online, and there has been a well-documented increase in the number of threats to data integrity due to spam and imposter participants [65]. However, many of these issues arose after the COVID-19 pandemic, and the current study was conducted in Spring 2019. Another consideration is that this study relies on self-report, which may limit clinical accuracy and may not fully capture autistic experiences, particularly for individuals with language difficulties or intellectual disabilities. Furthermore, the cross-sectional study design does not allow for causal inferences to be made [66]. Another point of consideration is that while our analytic approach allowed for detailed comparisons across gender and age subgroups, it did not allow for exploration of the interaction between multiple covariates in a single model. We opted to use our preregistered set of analyses as it allowed for new information to be gained separately about whether gender and age are risk factors in increased suicidality among autistic people in midlife and old age. This could help inform how risk is assessed, and support is tailored towards older autistic people seeking mental health care. Our choice of analysis involves more steps than multivariate modelling (i.e., increasing risk of Type I errors), but preserves statistical power (our power calculations indicate that 54 participants were required per group to maintain 95% statistical power, which our sample exceeds in both gender and age group analyses); we caution readers to consider this when interpreting our findings. We recommend that future studies with larger samples may benefit from using multivariate modelling to explore whether autism, gender, and age interact in suicidality. Additionally, the current study also explored gender differences between men and women, with gender diverse participants being excluded from analyses. This decision was made due to pragmatic reasons, as only 4 gender diverse participants took part. Gender diverse people have been found to be at high risk of self-harm and suicidality in the general population [64], with autistic gender diverse people being found to experience barriers to appropriate healthcare [67]. As such, future research should explore the self-harm and suicidality experiences of gender diverse autistic people. And finally, potential survivor bias may skew data by predominantly representing healthier older adults and excluding critical insights from individuals who died by suicide that can only be gained through mortality reports [68, 69]. While these limitations may restrict the overall generalisability of the findings, the results offer valuable insights into self-reported self-harm and suicidality experiences of autistic adults in midlife and old age. This study represents a critical step towards an enhanced understanding of suicidality in ageing autistic populations and highlights the need for tailored support to mitigate the risk of suicide in this population.

## Conclusion

This study provides important clinical insights regarding patterns in suicidality and suicidal behaviours in middle-aged and older autistic adults. First, middle-aged and older autistic adults demonstrate significantly higher rates of self-harm and suicidal behaviours compared to non-autistic peers. Second, notable gender differences exist, with autistic women showing heightened risk across all suicidality and suicidal self-harm items, highlighting the need for gender-sensitive approaches in assessment and intervention. Third, our findings emphasise the persistence and exacerbation of suicide risk into midlife and old age, with older autistic adults reporting higher rates of lifetime and recent suicidality and suicidal self-harm behaviours, underlining the need for life-course approaches to suicide prevention. Taken as a whole, our findings represent a significant contribution to our understanding of the rates of suicidality in autistic adults. Moving forward, it is crucial to consider gender- and age-related differences in future suicidality research, and to also consider potential mechanisms underpinning suicidality in autistic populations. By doing this, clinicians will be better equipped to support autistic people, including those in midlife and old age.

## Electronic supplementary material

Below is the link to the electronic supplementary material.

Supplementary Material 1

## Data Availability

The data that support the findings of this study are held by Dr. Gavin Stewart, but the availability of these data is restricted. The data were used under license for the current study and are not publicly available. However, the data may be available from the authors upon reasonable request and with permission from Dr. Gavin Stewart.

## Abbreviations

RAADS-14: Ritvo Autism and Aspergers Diagnostic Scale, a measure of autistic traits
PHQ-8/9: Patient Health Questionnaire, a measure of depression
ANOVA: Analysis of Variance, statistical test to analyse difference between means of two or more groups
