# Supplementary material for: Self-harm and suicidality experiences of autistic and non-autistic adults in midlife and old age
Source: Mol Autism. 2025 Nov 25;16:58. doi: 10.1186/s13229-025-00693-x (PMC12648868; doi:10.1186/s13229-025-00693-x)
Supplement: Supplementary file 1 — Supplementary Material 1 [file 13229_2025_693_MOESM1_ESM.docx]

| **Supplementary Table 1.** *Within-Group Gender Differences in Prevalence Rates of Self-harm and Suicidality.* | | | | | | |
| --- | --- | --- | --- | --- | --- | --- |
|  | **Autistic**  **Men**  **(n=98)** | **Autistic**  **Women**  **(n=124)** | **Non-Autistic Men**  **(n=84)** | **Non-Autistic**  **Women**  **(n=82)** | **Group**  **Difference** | **Effect**  **Size** |
| **Suicidal Ideation - Many people have felt that life is not worth living. Have you felt that way?** | | | | | | |
| No | 31 (32.0%) | 28 (22.6%) | 68 (81.0%)^ǂ^ | 55 (67.5%)^ǂ^ | Autistic: χ^2^=9.78, *p*=.008**  Non-Autistic: χ^2^=8.80, *p=*.012* | Autistic: *v=*.21  Non-Autistic: *v=*.23 |
| Yes, once | 18 (18.6%)^ǂ^ | 11 (8.9%)^ǂ^ | 16 (19.0%) | 20 (24.4%) |  |  |
| Yes, more than once | 46 (48.4%)^ǂ^ | 85 (68.5%)^ǂ^ | 0 (0.0%)^ǂ^ | 7 (8.5%)^ǂ^ |  |  |
| **Thoughts of Harm - Have you contemplated harming yourself (for example by cutting, biting, hitting yourself or taking an overdose)?** | | | | | | |
| No | 41 (43.6%)^ǂ^ | 32 (25.8%)^ǂ^ | 68 (81.0%) | 56 (68.3%) | Autistic: χ^2^=9.64, *p*=.008**  Non-Autistic: χ^2^=3.73, *p=*.155^n/s^ | Autistic: *v=*.21  Non-Autistic: *v=*.15 |
| Yes, once | 14 (14.9%) | 15 (12.1%) | 11 (13.1%) | 16 (19.2%) |  |  |
| Yes, more than once | 39 (41.5%)^ǂ^ | 77 (62.1%)^ǂ^ | 5 (6.0%) | 10 (12.2%) |  |  |
| **Thoughts of Harm (Recent) - Have you contemplated harming yourself in the last 12 months?** | | | | | | |
| No | 52 (55.3%) | 71 (57.3%) | 81 (96.4%) | 76 (92.7%) | Autistic: χ^2^=0.08, *p*=.775^n/s^  Non-Autistic: χ^2^=1.14, *p=*.287^n/s^ | Autistic: *v=*.02  Non-Autistic: *v=*.08 |
| Yes | 42 (44.7%) | 53 (42.7%) | 3 (3.6%) | 6 (7.3%) |  |  |
| **Any Deliberate Harm - Have you deliberately harmed yourself, whether or not you meant to end your life?** | | | | | | |
| No | 52 (55.3%) | 47 (37.9%) | 76 (90.5%) | 68 (82.9%) | Autistic: χ^2^=6.54, *p*=.011*  Non-Autistic: χ^2^=2.06, *p=*.152^n/s^ | Autistic: *v=*.17  Non-Autistic: *v=.*11 |
| Yes | 42 (44.7%) | 77 (62.1%) | 8 (9.5%) | 14 (17.1%) |  |  |
| **Frequency of Harm – If yes, how many times have you harmed yourself?** | | | | | | |
| Once | 4 (9.5%) | 14 (17.7%) | 5 (62.5%) | 6 (42.9%) | Autistic: χ^2^=1.47, *p*=.479^n/s^  Non-Autistic: χ^2^=0.99, *p=*.608^n/s^ | Autistic: *v=*.08  Non-Autistic: *v=*.21 |
| Twice | 5 (11.9%) | 8 (10.1%) | 2 (25.0%) | 4 (28.6%) |  |  |
| Three or more times | 33 (78.6%) | 57 (72.2%) | 1 (12.5%) | 4 (28.6%) |  |  |
| **Any Deliberate Harm (Recent) - Have you harmed yourself in the last 12 months, whether or not you meant to end your life?** | | | | | | |
| No | 76 (80.9%) | 105 (84.7%) | 83 (98.8%) | 77 (93.9%) | Autistic: χ^2^=0.56, *p*=.456^n/s^  Non-Autistic: χ^2^=2.87, *p=*.090^n/s^ | Autistic: *v=*.05  Non-Autistic: *v=*.13 |
| Yes | 18 (19.1%) | 19 (15.3%) | 1 (1.2%) | 5 (6.1%) |  |  |
| **Suicidal Harm - Have you deliberately taken an overdose or harmed yourself with the intention to end your life?** | | | | | | |
| No | 83 (88.3%) | 91 (73.4%) | 82 (97.6%) | 78 (95.1%) | Autistic: χ^2^=7.38, *p*=.007**  Non-Autistic: χ^2^=0.74, *p=*.389^n/s^ | Autistic: *v=*.18  Non-Autistic: *v=*.07 |
| Yes | 11 (11.7%) | 33 (26.6%) | 2 (2.4%) | 4 (4.9%) |  |  |
| *Note.* ^ǂ^ Adjusted residual indicates proportional difference. **p* <.05, ***p* <.01. ****p* <.001. Linear regressions were used to account for depression in analyses;  Suicidal Ideation: R^2^=.362, F=74.34***, depression *b*=.249, autism group =.399, gender *b*=.116.  Thoughts of Harm: R^2^=.295, F=54.74***, depression *b*=.307, autism group =.270, gender *b*=.138.  Thoughts of Harm (recent): R^2^=.265, F=47.01***, depression *b*=.372, autism group =.203, gender *b*=.001^n/s^.  Deliberate Harm: R^2^=.297, F=55.10***, depression *b*=.367, autism group =.208, gender *b*=.113.  Deliberate Harm (recent): R^2^=.114, F=16.75***, depression *b*=.319, autism group =.023, gender *b*=.036 ^n/s^.  Suicidal Harm: R^2^=.119, F=17.71***, depression *b*=.320, autism group =.106, gender *b*=.138. | | | | | | |

| *Supplementary Table 2. Gender differences in self-reported types of harm used by those who have self-harmed in the autistic group.* | | | | |
| --- | --- | --- | --- | --- |
|  | **Autistic Men**  **(n=42)** | **Autistic Women**  **(n=71)** | **Group Difference** | **Effect Size** |
| **Types of Harm – Have you done any of the following to harm or endanger yourself?** | | | | |
| Stopping prescribed medication | 11 (26.2%) | 10 (13.5%) | χ^2^=2.90,  *p*=.088^n/s^ | *v*=.16 |
| Swallowing dangerous objects or products | 13 (31.0%) | 14 (18.2%) | χ^2^=2.53,  *p*=.112^n/s^ | *v*=.15 |
| Ingesting alcohol or recreational/illicit drug | 13 (31.0%) | 15 (19.7%) | χ^2^=1.88,  *p*=.170^n/s^ | *v*=.13 |
| Ingesting a medication more than the normal dosage | 23 (54.8%) | 36 (46.8%) | χ^2^=0.70,  *p*=.404^n/s^ | *v*=.08 |
| Self-injury (e.g., cutting, scratching, hitting) | 28 (66.7%) | 46 (59.0%) | χ^2^=0.68,  *p*=.408^n/s^ | *v*=.08 |
| *Note. Participants could select multiple options, thus total does not equal 100%.* | | | | |

| *Supplementary Table 3. Self-reported prevalence rates of self-harm and suicidality in the Autistic group, split into midlife (age 40-64 years) and old age (65+ years).* | | | | |
| --- | --- | --- | --- | --- |
|  | **Autistic**  **Midlife group (n=131)** | **Autistic**  **Old Age group (n=88)** | **Group Difference** | **Effect Size** |
| **Suicidal Ideation - Many people have felt that life is not worth living. Have you felt that way?** | | | | |
| No | 41 (31.3%) | 18 (20.5%) | χ^2^=3.264,  *p=*.162^n/s^ | *v*=.13 |
| Yes, once | 18 (13.7%) | 11 (12.5%) |  |  |
| Yes, more than once | 72 (55.0%) | 59 (67.0%) |  |  |
| **Thoughts of Harm - Have you contemplated harming yourself (for example by cutting, biting, hitting yourself or taking an overdose)?** | | | | |
| No | 52 (40.0%)^ǂ^ | 21 (23.9%)^ǂ^ | χ^2^=6.20,  *p=*.043* | *v*=.17 |
| Yes, once | 15 (11.5%) | 14 (15.9%) |  |  |
| Yes, more than once | 63 (48.5%)^ǂ^ | 53 (60.2%)^ǂ^ |  |  |
| **Thoughts of Harm (Recent) - Have you contemplated harming yourself in the last 12 months?** | | | | |
| No | 88 (67.7%) | 35 (39.8%) | χ^2^=16.64,  *p*<.001*** | *v*=.27 |
| Yes | 42 (32.3%) | 53 (60.2%) |  |  |
| **Any Deliberate Harm - Have you deliberately harmed yourself, whether or not you meant to end your life?** | | | | |
| No | 73 (56.2%) | 26 (29.5%) | χ^2^=14.98,  *p*<.001*** | *v*=.26 |
| Yes | 57 (43.8%) | 64 (70.5%) |  |  |
| **Frequency of Harm – If yes, how many times have you harmed yourself?** | | | | |
| Once | 11 (19.3%) | 7 (10.9%) | χ^2^=1.68,  *p*=.432^n/s^ | *v*=.11 |
| Twice | 6 (10.5%) | 7 (10.9%) |  |  |
| Three or more times | 40 (70.2%) | 50 (78.1%) |  |  |
| **Any Deliberate Harm (Recent) - Have you harmed yourself in the last 12 months, whether or not you meant to end your life?** | | | | |
| No | 114 (87.7%) | 67 (76.1%) | χ^2^=4.97,  *p*=.026* | *v*=.15 |
| Yes | 16 (12.3%) | 21 (23.9%) |  |  |
| **Suicidal Harm - Have you deliberately taken an overdose or harmed yourself with the intention to end your life?** | | | | |
| No | 111 (85.4%) | 63 (71.6%) | χ^2^=6.20,  *p*=.013* | *v*=.17 |
| Yes | 19 (14.6%) | 25 (28.4%) |  |  |
| *Note.* ^ǂ^ Adjusted residual indicates proportional difference. **p* <.05, ***p* <.01. ****p* <.001. Linear regressions were used to account for depression in analyses;  Thoughts of Harm: R^2^=.143, F=13.63***, depression *b*=.289, age group =-.191.  Thoughts of Harm (recent): R^2^=.178, F=17.68***, depression *b*=.423, age group =.002^n/s^.  Deliberate Harm: R^2^=.288, F=52.83***, depression *b*=.359, age group =.094.  Deliberate Harm (recent): R^2^=.113, F=16.58***, depression *b*=.318, age group =.016^n/s^.  Suicidal Harm: R^2^=.107, F=15.58***, depression *b*=.224, age group =.077. | | | | |

| *Supplementary Table 4. Age differences in self-reported types of harm used by those who have self-harmed in the autistic group.* | | | | |
| --- | --- | --- | --- | --- |
|  | **Autistic**  **Midlife group (n=57)** | **Autistic**  **Old Age group (n=65)** | **Group Difference** | **Effect Size** |
| **Types of Harm – Have you done any of the following to harm or endanger yourself?** | | | | |
| Stopping prescribed medication | 13 (22.8%) | 8 (13.6%) | χ^2^=1.67,  *p*=.196^n/s^ | *v*=.12 |
| Swallowing dangerous objects or products | 8 (14.0%) | 19 (30.6%) | χ^2^=4.67,  *p*=.031* | *v*=.20 |
| Ingesting alcohol or recreational/illicit drug | 15 (26.3%) | 13 (21.3%) | χ^2^=0.41,  *p*=.523^n/s^ | *v*=.06 |
| Ingesting a medication more than the normal dosage | 22 (38.6%) | 37 (59.7%) | χ^2^=5.28,  *p*=.0.22* | *v*=.21 |
| Self-injury (e.g., cutting, scratching, hitting) | 43 (75.4%) | 31 (49.2%) | χ^2^=8.71,  *p=*.003** | *v*=.27 |
| *Note. Participants could select multiple options, thus total does not equal 100%.* ****p* <.001 | | | | |

**APPENDICES**

**Appendix A.** *UK BioBank Self-Harm and Suicidality 8-item Questionnaire.*

Consent

[ ] Continue to the questionnaire exploring self-harm and suicidality

[ ] Skip to the next section

**Suicidal Ideation**

1. Many people have thoughts that life is not worth living. Have you felt that way?

[ ] No

[ ] Yes, once

[ ] Yes, more than once

[ ] I prefer not to answer (this will skip the rest of the questions on self-harm and suicidality)

**Self-Harming Thoughts**

1. Have you contemplated harming yourself, for example, by cutting, biting, hitting yourself, or taking an overdose?

[ ] No

[ ] Yes, once

[ ] Yes, more than once

[ ] I prefer not to answer (this will skip the rest of the questions on self-harm and suicidality)

1. Have you contemplated harming yourself in the last 12 months?

[ ] No

[ ] Yes

**Any Deliberate Self-Harm**

1. Have you deliberately harmed yourself, whether or not you meant to end your life?

[ ] No

[ ] Yes

1. (If Yes) How many times have you harmed yourself?

[ ] Once

[ ] Twice

[ ] Three or more times

1. Have you harmed yourself in the last 12 months, whether or not you meant to end your life?

[ ] No

[ ] Yes

1. Have you done any of the following to harm or endanger yourself?

[ ] Self-injury (e.g., cutting, scratching, hitting)

[ ] Ingesting a medication in excess of the normal dosage

[ ] Ingesting alcohol or a recreational or illicit drug

[ ] Swallowing dangerous objects or products

[ ] Stopping prescribed medication

[ ] None of the above

[ ] I prefer not to answer (this will skip the rest of the questions on self-harm and suicidality)

**Suicidal Self-Harm**

1. Have you deliberately taken an overdose with the intention to end your life?

[ ] No

[ ] Yes
